# Positioning women's and children's health in African union policy-making: a policy analysis

**DOI:** 10.1186/1744-8603-8-3

**Published:** 2012-02-16

**Authors:** Kadidiatou Toure, Rotimi Sankore, Shyama Kuruvilla, Elisa Scolaro, Flavia Bustreo, Babatunde Osotimehin

**Affiliations:** 1 Partnership for Maternal, Newborn & Child Health, The Secretariat hosted by WHO, 20, Avenue Appia, CH-1211 Geneva 27, Switzerland; 2 Africa Public Health Alliance & 15%+ Campaign, 14 Akintan Street, Dideolu Est, Ogba, Lagos Nigeria; 3 Family, Women's and Children's Health, World Health Organization, 20, Avenue Appia, CH-1211 Geneva 27, Switzerland; 4 United Nations Population Fund, 605 Third Avenue, New York, New York 10158, USA

**Keywords:** African Union, Millennium Development Goals (MDGs), policy-making, women's and children's health

## Abstract

**Background:**

With limited time to achieve the Millennium Development Goals, progress towards improving women's and children's health needs to be accelerated. With Africa accounting for over half of the world's maternal and child deaths, the African Union (AU) has a critical role in prioritizing related policies and catalysing required investments and action. In this paper, the authors assess the evolution of African Union policies related to women's and children's health, and analyze how these policies are prioritized and framed.

**Methods:**

The main method used in this policy analysis was a document review of all African Union policies developed from 1963 to 2010, focusing specifically on policies that explicitly mention health. The findings from this document review were discussed with key actors to identify policy implications.

**Results:**

With over 220 policies in total, peace and security is the most common AU policy topic. Social affairs and other development issues became more prominent in the 1990s. The number of policies that mentioned health rose steadily over the years (with 1 policy mentioning health in 1963 to 7 in 2010).

This change was catalysed by factors such as: a favourable shift in AU priorities and systems towards development issues, spurred by the transition from the Organization of African Unity to the African Union; the mandate of the African Commission on Human and People's Rights; health-related advocacy initiatives, such as the Campaign for the Accelerated Reduction of Maternal Mortality in Africa (CARMMA); action and accountability requirements arising from international human rights treaties, the Millennium Development Goals (MDGs), and new health-funding mechanisms, such as the Global Fund to Fight AIDS, Tuberculosis and Malaria.

Prioritization of women's and children's health issues in AU policies has been framed primarily by human rights, advocacy and accountability considerations, more by economic and health frames looking at investments and impact. AU policies related to reproductive, maternal, newborn and child health also use fewer policy frames than do AU policies related to HIV/AIDS, tuberculosis and malaria.

**Conclusion:**

We suggest that more effective prioritization of women's and children's health in African Union policies would be supported by widening the range of policy frames used (notably health and economic) and strengthening the evidence base of all policy frames used. In addition, we suggest it would be beneficial if the partner groups advocating for women's and children's health were multi-stakeholder, and included, for instance, health care professionals, regional institutions, parliamentarians, the media, academia, NGOs, development partners and the public and private sectors.

## Background

With limited time remaining to achieve the Millennium Development Goals (MDGs), there needs to be accelerated progress to achieve MDGs 4 and 5 and improve women's and children's health [1]. This is particularly true in Sub-Saharan Africa, where half of the 7.6 million annual under-five deaths and the over half of the 358 000 annual maternal deaths occur. Sub-Saharan Africa remains the region with the highest maternal mortality rate, with women having a 1 in 31 chance of dying from a pregnancy related cause; and is the region that marks the slowest progress towards the achievement of MDGs 4 and 5 [2,3].

African countries have a leadership role in developing and implementing the required policies and programmes to achieve progress in women's and children's health. However, many countries are faced with multiple development priorities and limited resources. Improving the health of women and children will require national commitment to the health agenda at the highest level.

Regional bodies such as the African Union (AU) set regional policy priorities. They also increasingly influence national and global policies as the need for linking global and national initiatives and for partner coordination increases [4].

The African Union has already shaped national health priorities. For instance, in 1999, the African Union became the first and only regional body to pass a charter on the rights of the child. In 2006, the Protocol to the African Charter of Human and People's Rights on the Rights of Women in Africa became the first convention to mandate state provision of comprehensive reproductive and sexual health services [5]. This resulted in the expansion of the legal grounds for abortion in many African countries and a fall in unsafe abortion rates [6]. Between 1997 and 2009, Benin, Chad, Ethiopia, Guinea, Mali, Niger, Swaziland and Togo all expanded the grounds on which abortion is legal [7].

African Union policies have also been instrumental in persuading some African policy-makers to recognize HIV/AIDS [8], leading to the establishment of HIV/AIDS commissions in all African countries. These commissions may have played a role in reducing the annual total of new HIV infections, which fell from 2.3 to 1.9 million from 2001 to 2008 [9].

As with HIV/AIDs and other issues, the African Union can be an important stakeholder in promoting commitments to improve the health of women and children and ensuring accountability for their implementation. The Campaign for the Accelerated Reduction of Maternal Mortality in Africa, led by the African Union, has been launched in 34 countries [10]. In 2010, the African Union Summit of Heads of States held its first Assembly on the theme of maternal, infant and child health and development in Africa [11]. The declaration emanating from this Summit has spurred various commitments by member states to women's and children's health and related resolutions in the Pan African Parliament [12,13].

This paper analyses how the African Union has prioritized policy-making on women's and children's health, and outlines the implications of the trends and how policy-making can be strengthened.

## Methods

The main method used in this policy analysis was a document review of all African Union policies developed from 1963 to 2010, focusing specifically on policies that explicitly mentioned health. The findings from this document review were discussed with key experts to identify policy implications.

### Document review

To conduct the document review, all African Union Head of State Assembly Outcome Documents were obtained from the African Union online archive [14]. "Outcome documents" are defined as the overarching policy documents adopted by any African Union Summit of Heads of State and Government. Each outcome document contains various decisions, resolutions and declarations, which are referred to as policies. There were a total of 55 Outcome Documents with a subset of 884 policies.

The 884 policies found in the 55 outcome documents were categorized according to the 8 portfolios of the African Union Commission (Table 1). Policies were categorized based on the specific topics contained within these AU portfolios. To categorize all policies, two additional categories were created, which were not African Union portfolios *per se*: one for African Union governance and one for cross-cutting policies. The combined list of the eight African Union portfolios and the two additional categories are (in alphabetical order):

**Table 1:** African Union Policy Categories 1963-2010 (in descending order)

Policy categories	1963-1970		1971-1980		1981-1990		1991-2000		2001-2010		Total	
Peace and security	32	42%	38	43%	43	36%	35	24%	73	16%	**221**	**25%**

Political affairs	10	13%	7	8%	15	13%	27	19%	93	20%	**152**	**17%**

African Union governance	24	32%	10	11%	19	16%	8	6%	67	15%	**128**	**14%**

Social affairs	2	3%	8	9%	10	8%	21	15%	61	13%	**102**	**12%**

Economic affairs	2	3%	4	5%	22	18%	25	17%	48	11%	**101**	**11%**

Cross-cutting policies	3	4%	10	11%	4	3%	10	7%	48	11%	**75**	**8%**

Rural economy, agriculture	0	0%	2	2%	2	2%	5	3%	30	7%	**39**	**4%**

Human resources, science, technology	3	4%	3	3%	2	2%	6	4%	17	4%	**31**	**4%**

Trade and industry	0	0%	2	2%	2	2%	6	4%	15	3%	**25**	**3%**

Infrastructure and energy	0	0%	4	5%	1	1%	0	0%	5	1%	**10**	**1%**

**Total**	**76**	**100%**	**88**	**100%**	**120**	**100%**	**143**	**100%**	**457**	**100%**	**884**	**100%**

1. African Union Governance: Core Business Management, Development of Strategies, Implementation Facilitation, Costs, Appointments, New Structures.

2. Cross-cutting: Development, Poverty Reduction, Regional Cooperation, and any declarations that cover issues in more than one category.

3. Economic affairs: Economic Integration, Monetary Affairs, Private Sector Development, Investment and Resource Mobilisation.

4. Human resources, science and technology: Education, Information and Communication Technology, Youth, Science and Technology, Human Resources.

5. Infrastructure and energy: Energy, Transport, Communications, Infrastructure and Tourism.

6. Peace and security: Conflict Prevention, Management and Resolution, and Terrorism Issues.

7. Political affairs: Democracy, Human Rights, Good Governance, Electoral Institutions, Humanitarian Affairs, Civil society Organisations, Refugee Matters.

8. Rural economy and agriculture: Rural Economy, Agriculture and Food Security, Livestock, Environment, Water, Natural Resources and Desertification

9. Social affairs: Health, Children, Drug Control, Gender, Labour and Employment, Sports and Culture, Migration

10. Trade and industry: Trade, Industry, Customs and Immigration Matters

The numbers of policies developed in each of these categories were compared across time to determine historical trends in AU policy-making.

In order to identify health-related policies, a full text keyword search of the 55 outcome documents using the term "health" was conducted. This search yielded 56 policies from across all portfolios. One of these policies was excluded as the reference was to 'healthy management' practices and not to health *per se*, resulting in a total of 55 health policies that were included in this analysis.

Some specific health policies-for example on Polio or HIV/AIDS-were not captured in the keyword search using "health". Traditionally all health-related policies are located within the Social Affairs portfolio. To ensure that all policies related to health were captured, the authors further reviewed and categorized the 102 policies within the Social Affairs portfolio based on their reference to specific health topics e.g. AIDS/TB/Malaria, reproductive, maternal, newborn and child health (RMNCH), and to women's and children's rights to health and development (Table 2). An additional 25 health-related policies were identified.

**Table 2:** Number of African Union Social Affairs Policies, by Category 1963-2010, (in descending order)

	1963-1970	1971-1980	1981-1990	1991-2000	2001-2010	Total
Other social affairs	1	7	6	3	28	**45**

Women's and children's health	0	2	3	2	20	**27**

AIDS/TB/Malaria	0	0	0	11	10	**21**

Other health	1	0	1	4	3	**9**

**Total**	**2**	**9**	**10**	**20**	**61**	**102**

A total of 81 AU health-related policies were then included in the document review (Annex 1-list of policies).

In order to identify how and why women's and children's health is prioritized in African Union policies, the authors analysed all health-related policies, according to the main arguments or "frames" used. Based on a review of previous policy analyses [15-22] a preliminary list of policy frames was identified. For analysis, these frames were defined based on the topics covered (e.g. costs, economic growth, or budgets would relate to an economic frame), type of evidence used (e.g. health outcomes, mortality rates etc., relate to a health frame), and emphasis (e.g. calls to action and participation in events relate to an advocacy frame) etc... These frames were further defined in an iterative process of developing, testing and refining the analytical framework for the document review. The final frames used in the document review are listed below (in alphabetical order):

1. **Accountability**: legal, policy, and monitoring requirements, including human rights, and African Union and Millennium Development Goals reporting.

2. **Advocacy**: prioritization statements from influential stakeholders, media coverage, events, and advocacy campaigns.

3. **Economic**: social and economic development, including trade, productivity, cost-effectiveness analysis, efficiency and trade-offs.

4. **Health**: scientific evidence and technical information on health outcomes, effective interventions and health systems.

5. **Other**: anything falling outside of frames 1-4, such as cultural norms and references.

In additional to these frames, and in line with Walt's framework of policy analysis, which promotes a review of content and context of policies, as well as a review of actors and processes that influence policy-making [23], the coders noted key actors involved, related events, implementation and accountability mechanisms and recommended actions.

Using these coding parameters, two analysts first coded a random sample of 5 of the 81 health-related policies. The coding was done in Excel. The inter-rater reliability was assessed, and through an iterative process, the analysts identified discrepancies in coding, refined the analytical frames in discussion with the research team and retested inter-rater reliability. The final intercoder agreement was 0.80, which is considered acceptable [24].

The authors also compared the policy frames used in AU policies related to MDGs 4 and 5 on reproductive, maternal and child health, with AU policies on MDG 6 related to HIV/AIDS, tuberculosis and malaria.

### Discussions with key actors on policy implications of the findings of the document review

To validate the findings and discuss policy implications, results of the document review were discussed with a purposive sample of 10 key actors involved in the African Union and in health policy and processes on the continent.

The actors included high-level representatives of the African Union, ministries of health and non-governmental organizations working on African Union issues.

Interviews were conducted in a semi-structured format with written notes (Annex 2-list of questions). Discussions included a report by the interviewer of the preliminary findings of the document review and information from key actors on the reasons underlying review findings, policy implications of these findings and recommendations for how, based on results, women's and children's health could be further prioritized.

Participating experts were informed of the intent and purpose of this research and were informed that their views would help shape the content of this policy analysis. They were made aware that the interviews would guide the direction of the document and would be referenced anonymously in the paper. Oral consent was sought during the interview and the research paper was circulated to interviewees to ensure that content reflected the discussions.

### Limitations

This analysis has certain limitations. Because policies were identified using a health keyword search, a number of analysed policies may not have referred to health issues, i.e. policies nominating regional candidates for the position of Secretary General of the World Health Organization. As such the analysis of the 'other' policies that refer to health, mix policies on health issues, i.e. avian flu as well as policies on social determinants, as well as policies on political issues.

Additionally, the low number of discussions with experts to inform policy implications and recommendations and the fact that interviewed experts are all current office bearers represents a limitation, as their expertise did not expand over the full period of existence of the Organization of African Unity and African Union.

## Results

### Historical trends in African Union policy-making

**Figure 1:**
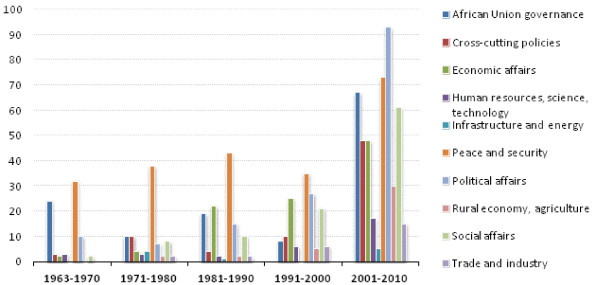
**Number of African Union Policies, by Category 1963-2010**.

Since 1963, 55 African Union summits resulted in a total of 884 policies. 25% of these policies focus on peace and security, 17% on political affairs, and 14% on African Union governance. Economic and social affairs policies account for 11% and 12% of all AU policies respectively (Figure 1 and Table 1).

Priorities in African Union policies have changed over time. Peace and security policies decreased from more than 40% of policies prior to 1980 to 16% in 2001-2010. The proportion of AU political affairs policies rose from 13% before 1970 to 20% in 2001-2010 (Figure 1 and Table 1).

Both economic and social affairs policies have increased substantially: from 3% prior to 1970 to 11% and 13% respectively between 2001 and 2010. However, the proportion of economic and social policies peaked between 1991 and 2000 and is now declining, while the number of rural economy and agriculture-related policies are increasing (Figure 1).

**Figure 2:**
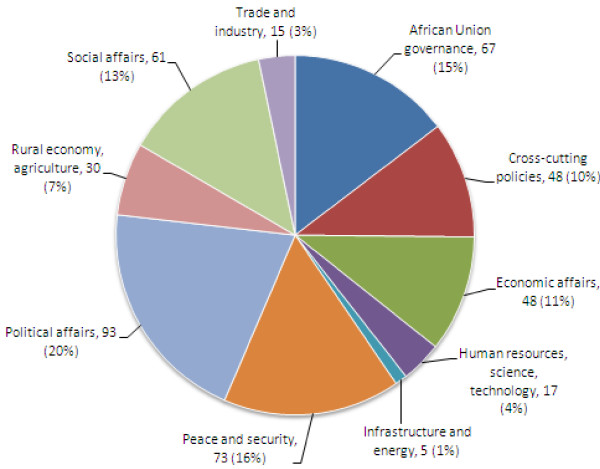
**Breakdown of African Union Policies, by Category 2001-2010**.

This trend analysis also notes a sharp increase in the number of policies adopted by assemblies after the shift from the Organization of African Unity to the African Union in 2002-52% of all African Union policies were adopted between 2001 and 2010 (more than the total number of policies passed in the preceding four decades) (Figure 2).

**Figure 3:**
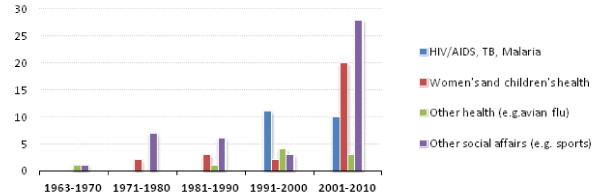
**Number of African Union Social Affairs Policies, by Category 1963-2010**.

A sharp increase in the number of health policies since 1991 has in part driven the increase in social affairs policies. Health accounted for 85% of social affairs policies between 1991 and 2000 and for 56% between 2001 and 2010 (Figure 3). The increase in the 1990s was due to a rise in the number of MDG 6-AIDS/TB/malaria-policies. Since 2000 there has been a similar increase in MDG 4 and 5-related policies on reproductive, maternal and child health. Other health policies-such as the 1987 Declaration on Health as a Foundation for Development and the 1991 Declaration on the current African Health Crisis-mostly occurred in the Organization's first three decades. They defined health as an African Union priority, and as a foundation for development and economic growth.

### Policy frames for women's and children's health in 81 policies that mention health

Women's and children's health policies use fewer frames (1.8 frames per policy) than do AIDS/TB/malaria-related policies (2.5 frames per policy). Within women's and children's health-related policies, human rights policies use the most frames to justify investments in women's and children's health and development. Overall, AIDS/TB/malaria policies use the health and economic frames slightly more often than women's and children's health policies (Table 3).

**Table 3:** Frames used in AU policies that mention health (in descending order)

	Women's and Children's Health		MDG 6		Other		Total
Procedural	20	42%	16	31%	14	25%	**50**

Advocacy	15	31%	17	33%	17	30%	**49**

Economic	6	13%	10	19%	21	37%	**37**

Health	7	15%	9	17%	5	9%	**21**

**Total**	**48**	**100%**	**52**	**100%**	**57**	**100%**	**157**

The frames used to prioritize women's and children's health based on our review of the 81 documents that mention health are now discussed in the following sections in alphabetical order.

#### Accountability frame

Women's and children's health is often framed using human rights, and the health of women and children is often addressed by rights policies. Between 2001 and 2010, there were 13 policies on women's and children's rights, empowerment and health, but there were only five policies specifically focused on Millennium Development Goals 4 and 5.

Rights policies address health issues related to women and children and define government obligations to provide health services. For example, the 2005 Protocol to the African Charter of Human and People's Rights on the Rights of Women in Africa addresses an array of gender-related health issues and other determinants of health. These include: access to education and food; child labour and forced labour; physical and sexual violence; harmful traditional practices; early marriage; reproductive health rights; maternal mortality; cancers; menopause; and mental disorders.

Legal, policy, and monitoring requirements-including human rights and African Union reporting-account for 42% of frames used by women's and children's health policies, 31% for AIDS/TB/malaria policies and 25% for other health policies.

#### Advocacy frame

Advocacy statements, policies and related activities constitute 33% of the frames used in AIDS/TB/malaria policies, and 31% of those used in women's and children's health policies. Advocacy statements have considerably increased in policies that refer to women's and children's health.

Referencing global goals and policies such as the Millennium Development Goals and regional events such as the World Summit on the Child has been significant. In the 2010 African Union Declaration on Maternal, Infant and Child Health and Development in Africa, member states:

"Individually and collectively reaffirm our previous commitments aimed at accelerating the health of our people and the social development of Africa. In this regard, we re-dedicate ourselves and commit our countries to accelerate efforts to improve the state of health of Africa's women and children and thereby attain all Millennium Development Goals (MDGs) particularly MDGs 4, 5 and 6 by 2015."

#### Health frame

Health frames include research, evidence and information on health outcomes and the burden of diseases. 17% of the frames used in AIDS/TB/malaria policies are health, 15% are in women's and children's health policies.

Generally in these policies, claims of the severity of the women's and children's health situation are not substantiated by scientific data.

#### Economic frame

Economic frames in policy include the use of arguments based on socioeconomic development, cost effectiveness and investment. Economic arguments constitute 13% of frames used in women's and children's health policies, 19% of those used in AIDS/TB/malaria policies and 37% of those used in other policies that mention health.

The use of the economic frame in women's and children's health policies is infrequent and unspecific. An example is seen in the 2008 Declaration on Maternal, Newborn and Child Health, which: *"Recognises with concern that poor maternal, infant and child health remains a major challenge confronting the continent and undermining its development efforts"*.

The bulk of policies that cite the contribution of women and children to development do not define or quantify it, or estimate the consequences of ill health. The closest numerical definition of the contribution of women to development is seen in the 1990 Resolution on the World Summit states that: *"African Women produce over 70% of the food crops in many parts of the Continent"*.

The cost-effectiveness of women's and children's health interventions is also rarely used as an argument.

## Discussion

### Policy implications of the document review

The document review yielded some findings that can be used to shape knowledge and advocacy efforts to prioritize and strengthen references to women's and children's health in African Union Policies.

The African Union predominantly views the health of women and children as a human right. This is seen in the inclusion of health entitlements in rights policies as well as in the referencing of rights policies in women's and children's health policies. Those organizations and individuals wishing to promote a post-2015 agenda for health can make use of the continued framing of women's and children's health in the rights discourse [25]. The rights discourse can also promote the implementation of essential services for women and children through the use of related accountability mechanisms such as the African Court on Human and People's Rights.

Accountability and monitoring requirements are heavily referenced in African Union policies, and receive substantial attention within the assemblies. These requirements provide an entry point for the issue to remain on the policy agenda. This use of the accountability may become more pertinent, especially as the recognition of the African Union increases globally, regionally and nationally. One public health expert notes that *"as the African Union becomes stronger, these policies will become more important because most of them incorporate monitoring and reporting clauses."*

Advocacy plays an important role in prioritizing issues. In part, this is due to the increased participation of advocates in African Union dialogue since the inception of the African Union in 2002. Advocacy for policy prioritization takes many shapes. For instance, prior to and during the July 2010 Summit, many articles appeared in Kampala and regional journals pointing to efforts undertaken by some countries to improve women's and children's health, highlighting the lack of progress in others and comparing regional health spending by all countries [26-28].

While the use of the advocacy frame has been successful it has been challenging for the women's and children's health community. As noted by a public health specialist:

"*Where the AIDS paradigm has a central message, and malaria has insecticide-treated nets*, [advocacy for] *reproductive, maternal, newborn and child health is complex. The messages range from coverage of different interventions along the continuum of care, to health systems issues and social determinants. When disaggregated, we note that a lot of them fall outside of the health sector. Grappling with the complex recommendations behind reproductive, maternal, newborn and child health has been challenging, and advocacy messages need to be simplified."*

The women's and children's health community should pursue its efforts in aligning and simplifying communication on key interventions to maximize campaign impact.

While scientific evidence on trends in Millennium Development Goals 4 and 5 and related interventions, policies, financing and practices [29] exists, it is rarely used to support policies on women's and children's health. Processes like the Countdown to 2015 provide evidence. Research institutes such as the Institute for Health Metrics and Evaluation, and development organizations such as the World Health Organization, United Nations Population Fund, World Bank and UNICEF, all provide substantial evidence on health outcomes and underlying causes [30,31].

Health advocates may choose to look at more effective ways to package, promote and disseminate these data to policy-makers in countries to ensure that policies are evidence-based. In addition to demonstrating that the implementation of affordable packages of interventions can save lives, they may also consider that this information will point to the severity of the women's and children's health crisis. As noted by one public health specialist:

*"The AIDS and malaria movements, using health outcomes and impact of these diseases on the societies, have managed to position these diseases as crises. While stakeholders acknowledge that maternal, newborn and child health is a problem, they are rarely aware of the magnitude of this problem, and this is because *[the health] *community has not positioned the issue using scientific communication. We have tabled our messages on emotional argumentation."*

In addition to providing scientific evidence on the health burden, its causes and remedies, increased effort to demonstrate the economic impact of women's and children's health on national and regional economies would help to prioritize this issue among Heads of States. This is reflected in the intervention of certain Heads of States during the 2010 Summit who noted that while the health of women and children is a cause for concern, the attention provided to the issue must be shared with other sectors that have a direct bearing on countries' growth and productivity [32].

Evidence of the productivity gains related to improved women's and children's health exists, though it is sparse and tends to reflect global figures. USAID estimates annual global losses in productivity due to maternal and newborn deaths at US$15 billion [33]. Regionally, the West African Health Organisation (WAHO) estimated in 2007 that static health investment from 2007 to 2015 in the countries of the Economic Community of West African States would result in a loss of US$5 billion [34].

The proven cost-effectiveness of women's and children's health interventions is also rarely used as an argument, although clear evidence to support investment does exist. For example, research has indicated that for every dollar spent on family planning, four dollars are saved on treatment for complications of unwanted pregnancies [35].

However, economic evidence varies greatly in quantity, quality and availability, which perhaps explains the restricted use of the economic frame. Traditional reluctance to justify health investment in economic terms may also be a factor. One health expert notes:

*"There has traditionally been some resistance by the health community to using economic arguments to justify health investments because of a reluctance to associate health spending to returns to the state. This trend is slowly subsiding and research on the impact of health investment is increasing*."

### Processes for improving prioritization of women's and children's health in African Union policy-making

#### Regional institutions and their roles in the prioritization process

Formal processes for prioritizing issues within African Union Assemblies include African Union Commission requests and recommendations; member state requests; and outcomes of regional ministerial conferences.

The African Union Commission aims to prioritize health issues based on member state interests and concerns (as defined by conferences of African ministers, member state requests and assemblies). This gives programmes legitimacy and encourages their implementation by countries. One expert notes the importance of this consultative process in the success of health campaigns resulting from initiatives such as the International Conference for Population and Development (ICPD). Another expert states the need to take account of local factors when pursuing globally agreed goals: *"One day when optimal processes have been put in place we will have reached global goals through continental mechanisms. CARMMA is one of these ideal processes."*

In addition to supporting the reflection of country priorities in the African Union agenda, the Commission plays a crucial role in defining it. The current Commissioner for Social Affairs has successfully positioned women's and children's health as a crucial issue for the continent, and has been the principal voice behind the regional campaign targeting its improvement. The Campaign for the Accelerated Reduction of Maternal Mortality in Africa (CARMMA) is an example of a successful campaign being pushed by the Commission. Having been launched at the highest political level in 34 countries in Africa (as of March 2011), CARMMA reflects successful national ownership and prioritization of women's and children's health [36].

The Commission also monitors the implementation of policies. However, the proliferation of policies and the limited personnel and resources of the African Union Commission make the implementation of these policies and efforts to hold stakeholders accountable, daunting tasks. The monitoring of implementation in countries is a particular challenge. One expert notes:

"African Union Declarations provide ministries of health with the leverage we need in our national, regional and international advocacy. However more needs to be done to ensure strong, compelling monitoring of the implementation of these policies in countries. Partners need to support the African Union so that it can systematically provide sound progress reports on the implementation."

Other regional institutions also contribute to prioritization of health on the continent. The Pan African Parliament, created in 2001, to ensure a representation of the voices of African populations in regional decision- making, intervened in favour of maternal, newborn and child health during the July 2010 Summit. While the body currently does not have legislative powers, it aspires "to evolve into an institution with full legislative powers, whose members are elected by universal adult suffrage" [37]. An assessment of the Parliament notes that adopting this model, which is already in existence in the European Parliament, could render the body much more effective.

Currently relegated to an advisory role, the body still contributes to regional prioritization. For instance, in October 2010, the Pan African Parliament Assembly adopted a motion adopting the Africa Parliamentary Policy and Budget Action Plan for Implementation of July 2010 AU Summit Decisions on Maternal, Newborn and Child Health and Development in Africa, and Partnership for Eradication of Mother to Child Transmission of HIV and AIDS [38]. In 2011, the Speakers of African Parliaments adopted Resolution on Declaration of Commitment for the Prioritization and Implementation of African Union Summit Decisions on Youth Development and Maternal, Newborn and Child Health [39].

Similarly, the African Court on Human and People's Rights has a strong health element to its work [40]. To date, 45 African countries [41] have ratified the charter on the rights of the child and 28 have ratified the protocol on the rights of women [42]. If more could be encouraged to ratify, this would strengthen the mechanisms behind the regional conventions and result in greater accountability.

The Africa Peer Review Mechanism (APRM) assesses progress on health through its socioeconomic development remit [43]. For example, the APRM 2010 Review of Lesotho expresses concern at the lack of progress on women's and children's health [44]. As of June 2009, the APRM counted 29 states and reports have already been undertaken in at least 12 countries.

#### Actor networks and advocacy efforts

Strong actor networks can generate, through advocacy efforts, increased attention for women's and children's health at both country and regional levels [45]. The results of this document review indicate that advocacy has indeed been a force behind the uptake of health policies since 2002.

For example, during the Summit, health campaigners, development partners and Ministers of Health and Finance were invited to debate health financing during a high-level side event on health financing. Advocates were also invited to speak alongside Heads of States in the plenary session and requests from advocates such as Professor Jeffrey Sachs, Director of The Earth Institute at Columbia University, were included in the Summit outcome document.

With advocacy playing such an important role, it is crucial to harness the efforts of women's and children's health advocates by developing multi-stakeholder actor networks that reflect the needs of member states, while also spanning the continuum of care for women's and children's health [46]. Until 2008, African Union policies on women's and children's health tackled issues vertically. By contrast, AIDS, tuberculosis and malaria have often been included in the same policy.

This division in policy topics across the continuum of care has also been seen in the actor networks. The first streamlined global advocacy efforts for women's and children's health began as recently as 2005, when three global partnerships merged: the Partnership for Safe Motherhood and Newborn Health; the Healthy Newborn Partnership; and the Child Survival Partnership.

Although major networks of this type do not yet exist at regional level, effective actor networks are emerging and contributing to priority setting and policy implementation. Civil society groups have long been recognized as key actors in advocacy, and the development of the Africa Maternal, Newborn and Child Health Coalition, a regional NGO network on women's and children's health, was an important outcome of the July 2010 Summit [47]. One expert notes:

"Coordinated partner action at the regional and country level is crucial to ensure the wide dissemination of Head of State commitments and accountability for results. NGO groups can be particularly helpful in this sense."

Health-care professional associations in Africa can also influence priority setting and the accountability of states to produce results, either directly or by raising awareness of key issues [48,49]. These bodies also have an important role in collecting good-quality health data for monitoring and accountability[50]. At the global level, associations operating along the continuum of care have begun to collaborate, spurred by the activities of organizations such as the International Federation of Gynaecology and Obstetrics [51].

Non-traditional actors are beginning to play a role in advocacy for women's and children's health, including parliamentarians [52] and the media. The latter are increasingly recognized for raising awareness, for example by communicating the commitments made by Heads of States, and by pressurizing governments to perform. As one expert notes:

"*The *[women's and children's health] *community must involve non-traditional stakeholders in the reshaping of advocacy messages and widen their scope of engagement."*

To optimise their effectiveness nationally and regionally, actor networks should coordinate their work and involve the widest range of possible stakeholders. The Global Strategy for Women's and Children's Health provides some direction on expanding the types of stakeholders involved in women's and children's health efforts and notes the roles to be played by each of these different partner constituencies [53]. The process around the development of Global Strategy also sought to integrate regionally-defined priorities. Efforts are now being conducted by partners to align the implementation of Global Strategy commitments with regional and national plans and strategies [54].

The Commission on Information and Accountability for Women's and Children's Health has also anchored its recommendations on principles of national sovereignty and has placed the country at the center of accountability processes. Its recommendations are based on principles of national leadership and ownership of results, strengthening countries' monitoring and evaluation capacity and reducing reporting burdens of countries [55].

Funding and resources are also key factors, because women's and children's health initiatives can be hampered by the lack of a funding mechanism. One expert notes:

*"Financing does impact policy prioritization ... in order to be most efficient and effective *[funding] *must come through a defined platform" *[56].

The benefits of sustained, coordinated funding are reflected in the prioritization of AIDS/TB/malaria in African Union policies. The Global Fund plays a major role here, contributing a high percentage of official development assistance and development assistance for health funding [57].

Global actors have recognized the need for similar sustained commitments, and a substantial increase in health investment to close the "funding gap" for women's and children's health [58,59]. Recent estimates of the additional funding needed for the period 2011-2015 in 49 low-income countries (33 of which are African) amounted to US$88 billion [60]. A significant amount of this was committed in September 2010 (US$40 billion) and 2011 by governments and other donors at the annual meetings held by the United Nations General-Secretary on the Global Strategy for Women's and Children's Health [61].

In 2007 and 2008, US$4.7 billion and US$5.4 billion were committed to women's and children's health activities in developing countries, respectively. These amounts reflect a 105% increase between 2003 and 2008 and improved prioritization of countries based on the burden of maternal and child deaths. However more remains to be done [62].

Despite these new high-level commitments and subscription to the principles outlined in the Paris Declaration and the Accra Agenda for Action [63,64], funding is still uncoordinated and often routed through bilateral channels [65,66]. Some stakeholders argue that streamlined funding mechanisms are needed. For instance, the African Union Heads of States (in their 2010 declaration) called on the Global Fund to "create a new window" to fund women's and children's health.

## Conclusion

This analysis demonstrates that in African Union policies, women's and children's health is prioritized using fewer frames than AIDS/TB/malaria. It also demonstrates that women's and children's health is most often framed using advocacy and accountability as opposed to health and economic arguments.

Prioritizing women's and children's health in African Union policies could be strengthened by 1) widening the range of frames used to discuss these issues, and 2) developing a stronger evidence base to support both currently-used and new frames.

The analysis also notes that regional institutions have and continue to play an important role in prioritizing women's and children's health. It identifies advocacy as an important mechanism to prioritize women's and children's health while ensuring links between global, regional and national priorities, and an alignment of partner efforts in reproductive, maternal, newborn and child health.

In order to improve the prioritization of women's and children's health within African Union policy-making, women's and children's health stakeholders could: 1) support the African Union Commission and other regional bodies such as the Pan African Parliament in the prioritization of women's and children's health in policies, 2) strengthen existing regional and national women's and children's health networks in part by widening the range of partners to include the private sector, media and parliamentarians, and 3) align global efforts and campaigns with nationally-and regionally-defined priorities.

### Using a wider range of policy frames and strengthening the evidence base

In an environment where different priorities compete for funding, women's and children's health policies could claim more attention by using a wider range of frames. As noted by Heads of States in their Summit debate on women's and children's health, resource constraint is a key barrier to health investment. Therefore, the use of all available evidence would help advocates to make a more effective case for prioritizing women's and children's health in African Union policies.

It seems likely that increased use of economic arguments would define women's and children's health as an investment, highlight the cost-effectiveness of health interventions and assist decision-makers in allocating scarce resources, by defining the synergies between investments in social determinants and health outcomes.

To inform policy decisions more effectively, national and regional institutions have the option of gathering region-specific evidence on the cost savings that result from effective interventions, and on the impact of better health on growth and productivity. In the interim, existing global estimates, and regional calculations based on disability adjusted life years and gender disaggregated information, are available to inform policy discourse. Regional institutions could also gather evidence on the impact of investment on social determinants of health. This would inform debate by decision-makers about how to invest in other sectors that have an impact on women's and children's health, such as water and sanitation, education and transport [67,68].

Increased use of health frames would also aid the case for greater prioritization of women's and children's health, by providing compelling, specific evidence for the effectiveness of proven interventions. Much of the evidence on health outcomes already exists, and is reported by international journals such as *The Lancet *and in processes such as Countdown to 2015.

### Strengthen regional institutions and actor networks, and improve partner coordination

The African Union Commission and related bodies such as the Pan African Parliament are key fora for advocacy in favour of women's and children's health. However, their resources (human capital and financial) are stretched, and they would benefit from increased capacity to engage fully in advocacy and follow-up at regional, sub-regional and national levels. Increasing the capacity of regional institutions can be done through the financing of health-related programs, specific projects and staff. Additionally, partners with wide country presence have the option of entering into joint planning with regional entities and acting as the implementing arms of regional agreements. For instance, partners could track a certain number of indicators to monitor progress on women's and children's health in countries which regional institutions can feed into their assemblies. These processes already exist but could benefit from being used more systematically and by a wider range of partners.

While women's and children's health actors exist and are quite active, they have tended to operate in silos and focus on specific issues. We suggest that stronger and better coordinated actor networks, focusing across the continuum of care, would generate more effective advocacy, and encourage policy-making and implementation. National and regional committees on women's and children's health could be expanded to include-in addition to representatives from the Ministries of Health, donors and international organizations, health care professionals, academics and NGOs-the private sector, parliamentarians and the media. Collaboration among these partners would align efforts for more efficiency, improve the effectiveness of outlined strategies and widen the scope of actions to improve health.

Women's and children's health actors also have the potential to facilitate better integration of national priorities in regional and global policies. For instance, the development of the Global Strategy sought to integrate perspectives from countries through consultations with country representatives. In the future, similar processes might use established regional networks such as the Africa Maternal, Newborn and Child Health NGO Network and processes such as African Union Ministerial meetings to ensure that global initiatives are based on national and regional priorities and recommendations.

### Policy-making for better health

Campaigners for improved women's and children's health in Africa still face significant challenges. These include the limited capacity of the African Union bodies, the imperfect integration of national, regional and global priorities, and the lack of a central mechanism for the funding of women's and children's health. However, as our paper suggests, there is great scope for the African Union, other regional institutions and actor networks to work together more closely to develop and prioritize policies that improve the health of women and children in Africa.

## Annex 1-List of African Union health policy documents analysed

### All African Union Policies that mention the word 'heath'-Keyword search-health

1. 1963 Health, Sanitation and Nutrition

2. 1979 Declaration on the Rights and Welfare of the African Child

3. 1985 Resolution on the Drawing up of a Programme of Assistance to Africa by UNESCO in the Fields of Scientific Research and Development

4. 1987 Declaration of Health as a Foundation for Development

5. 1987 Resolution on Universal Immunization in Africa

6. 1987 Resolution on the Reconstruction of Chad

7. 1987 Resolution on the Candidature of Professor Gottlieb Lobe Monessoko for the Post of Director General of WHO

8. 1990 Declaration of the Assembly of Heads of States and Government of the Organization of African Unity on the Political and Socio-Economic Situation in Africa and the Fundamental Changes Taking Place in the World

9. 1990 Resolution on the adoption of the African Charter on the rights and welfare of the African Child

10. 1990 Resolution on the World Summit on Children

11. 1991 Declaration of the Twenty Seventh Ordinary Session of the Assembly of Heads of States and Government on Employment in Africa

12. 1991 Declaration on the current African Health Crisis

13. 1992 Declaration on AIDS epidemic in Africa

14. 1992 Resolution on AIDS and Africa: an agenda for action

15. 1994 Tunis Declaration on AIDS and the Child in Africa

16. 1994 Preamble to the Declarations and Resolutions adopted by the 30th Ordinary Session of the Assembly of Heads of State and Government

17. 1995 Addis Ababa Declaration on the Dakar African Platform for Action on Women

18. 1995 Declaration on the African Plan of Action concerning the situation on Women in Africa in the context of Family Health

19. 1995 Resolution on Mobilization of Resources for Africa's Economic and Social Development

20. 1996 Yaoundé Declaration on Polio Eradication in Africa

21. 1996 Resolution on the Regular reporting of the Implementation status of OAU Declaration on HIV/AIDS

22. 1996 Resolution on Bioethics

23. 1997 Harare Declaration on Malaria Prevention and Control in the Context of African Economic Recovery and Development

24. 1998 Ouagadougou declaration

25. 1998 Decision: Malaria Prevention and Control within the context of Africa's Economic Recovery and Development

26. 1999 Decision on the "First Meeting of States Parties to the Convention on the Prohibition of the Use, Stockpiling, Production and Transfer of Anti-Personnel Mines and on their Destruction"

27. 2000 Lome Declaration

28. 2000 Lome Declaration on HIV/AIDs in Africa

29. 2000 Decision on proposal for the eradication of tsetse flies on the African continent

30. 2000 CSSCDA Solemn Declaration

31. 2001 Decision on the declaration of 2001-2010 as the decade for traditional medicine

32. 2001 Decision on the report on implementation of the PoA on the eradication of tsetse flies in Africa.

33. 2002 Decision on the control of Arterial Hypertension in Africa

34. 2002 Decision on the status report on Global Alliance for Vaccines and Immunization (GAVI)

35. 2003 Decision on promoting the development of sustainable cities and towns in Africa

36. 2003 Declaration on the fifth WTO Ministerial Conference

37. 2003 Maputo Declaration on Malaria, HIV/AIDS, Tuberculosis and Other Related Infectious Diseases (ORID)

38. 2004 Decision on the Implementation of the New Partnership for Africa's Development (NEPAD)

39. 2005 Decision on the Interim Report on HIV/AIDS, Tuberculosis, Malaria and Polio

40. 2005 Decision on the proposal on sickle-cell anaemia

41. 2005 Decision on the Report of the Commission on Accelerating Action for Child Survival and Development in Africa to meet the MDGs

42. 2005 Declaration on the Review of the Millennium Declaration and the Millennium Development Goals (MDGs)

43. 2006 Decision on the Linkage between Culture and Education

44. 2007 Decision on avian flu

45. 2007 Addis Ababa Declaration on Science Technology and Scientific Research for Development

46. 2008 Decision on promotion of maternal, infant and child health and development

47. 2008 Decision on the Report on the Promotion of Maternal, Infant and Child Health in Africa

48. 2008 Decision on the Progress Report on the Implementation of the Commitments of the May 2006 Abuja Special Summit on HIV/AIDS, Tuberculosis and Malaria (ATM)

49. 2009 Decision on the specialized technical committees

50. 2009 Decision on the Report of the Implementation Status of Decision on Promotion of Maternal, Infant and Child Health and Development in Africa

51. 2009 Decision on the Themes of the July 2009, January 2010 and July 2010 Sessions of the Assembly

52. 2010 Declaration on information and communication technologies in Africa: Challenges and prospects for development

53. 2010 Decision on the Five (5)-Year Review of the Abuja Call for Accelerated Action Towards Universal Access to HIV/AIDS, Tuberculosis and Malaria Services in Africa

54. 2010 Declaration on Actions on Maternal, Newborn and Child Health and Development in Africa By 2015

55. 2010 Decision on the partnership for the eradication of mother to child transmission of HIV/AIDS

56. 2010 Decision on the Report of Head of State and Government Orientation Committee on NEPAD

### Additional policies identified through a review of subset of policies in African Union Social Affairs department based on established department themes

1. 1992 Resolution on the Summit on the Economic Promotion of rural women presented by Senegal

2. 1997 Decision: Harare Declaration on Malaria Prevention and Control

3. 1998 Establishment of an African Fund for AIDS Control

4. 1999 Decision on the ILO Convention on the Banning of the Worst Forms of Child Labour and Immediate Action for their Elimination

5. 2000 Decision on the Report of African Summit on Roll-Back Malaria

6. 2000 Decision on the Holding of an African Summit on HIV/AIDS, Tuberculosis and other related infectious diseases

7. 2001 Decision on the African Summit on HIV/AIDS, Tuberculosis and other related infectious diseases

8. 2001 Decision on the Pan-African Forum on the future of Children

9. 2002 Decision on the report of the African Committee on the rights and welfare of the Child

10. 2003 Decision on the Appointment of Members of the African Commission on human and people's rights

11. 2003 Decision on the Draft Protocol to the African Charter on Human and People's Rights Relating to the Rights of Women

12. 2004 Decision on AIDS Watch Africa (AWA) and the Implementation of the Abuja and Maputo Declarations on Malaria, HIV/AIDS, Tuberculosis and Other Related Infectious Diseases in Africa

13. 2004 Decision on the International Centre for the Education of Girls and Women in Africa (CIEFFA)-Doc. Assembly/AU/11 (V) Add.1

14. 2004 Solemn Declaration on Gender Equality in Africa

15. 2005 Decision of the appointment of members of the African experts on the rights and welfare of the child

16. 2006 Decision on the Progress Report on AIDS Watch Africa (AWA)

17. 2006 Decision on the election of one member of the African committee on the rights and welfare of the child

18. 2006 Decision on Abuja Call for Accelerated Action Towards Universal Access to HIV/AIDS, Tuberculosis and Malaria (ATM) Services in Africa

19. 2006 Decision on the continental framework for harmonization of approaches among member states and integration of policies on human rights and people affected by HIV/AIDS in Africa

20. 2007 Decision of the reports on the implementation of the African Union solemn declaration on gender equality in Africa

21. 2008 Decision of the reports on the implementation of the African Union solemn declaration on gender equality in Africa

22. 2008 Decision of the appointment of members of the African experts on the rights and welfare of the child

23. 2009 Decision on the African women's decade

24. 2010 Decision on the Establishment of the Fund for African Women

25. 2010 Decision on the Appointment of Members of the African Committee of Experts on the Rights and Welfare of the Child

## Annex 2-Questions to interviewers

The interviewees were asked to comment on:

• The prioritization of women's and children's health in the African Union; whether this could be improved;

• Which mechanisms and actors have contributed to the prioritization or de-prioritization of women's and children's health; and how these same mechanisms and actors could improve prioritization.

• Their perception of the value placed on African Union policies by states and development partners, and how this value could be increased.

## List of abbreviations

APRM: Africa Peer Review Mechanism; AU: African Union; AWA: AIDS Watch Africa; CARMMA: Campaign for the Accelerated Reduction of Maternal Mortality in Africa; EU: European Union; GAVI: Global Alliance for Vaccines and Immunization; ICPD: International Conference for Population and Development; MDGs: Millennium Development Goals; OAU: Organization of African Unity; PMNCH: Partnership for Maternal, Newborn & Child Health; WAHO: West African Health Organisation.

## Competing interests

The authors declare that they have no competing interests.

## Authors' contributions

KT: Conducted research and coding and wrote different iterations of the research article. RS: Advised on content of the research and assisted in conducting key expert interviews. SK: Contributed to the analysis and writing process and content. ES: Coded policies using the coding framework and helped refine coding framework. FB: Contributed to the design, analysis and writing of the paper. BO: Guided development of the overall paper. All authors read and approved the final manuscript.
